# Bugs as Drugs: Therapeutic Microbes for the Prevention and Treatment of Disease

**DOI:** 10.3201/eid2507.190582

**Published:** 2019-07

**Authors:** Soumaya Zlitni, Ami S. Bhatt

**Affiliations:** Stanford University, Stanford, California, USA

**Keywords:** microbiome, probiotics, microbe, disease, health, bacteria, prevention, treatment

The role of microbes and the microbiome in various aspects of human health and disease is a subject of ongoing intense study. The complexity of microbial communities and their interactions with the host present exceptional challenges for conducting these investigations that will require multipronged strategies to resolve. As the field undergoes the much-needed transition beyond association studies to a mechanistic understanding of how microbes influence their hosts, manipulating commensal microbes and using them for diagnostics and therapeutic interventions is the logical next step. Because microbiome research studies are being published at an unprecedented rate, a reliable book that reviews the seminal work and knowledge gaps in this field is pressingly needed. In *Bugs as Drugs: Therapeutic Microbes for the Prevention and Treatment of Disease *(Figure), the authors address major themes in microbiome research, particularly focusing on therapeutic applications.

**Figure:**
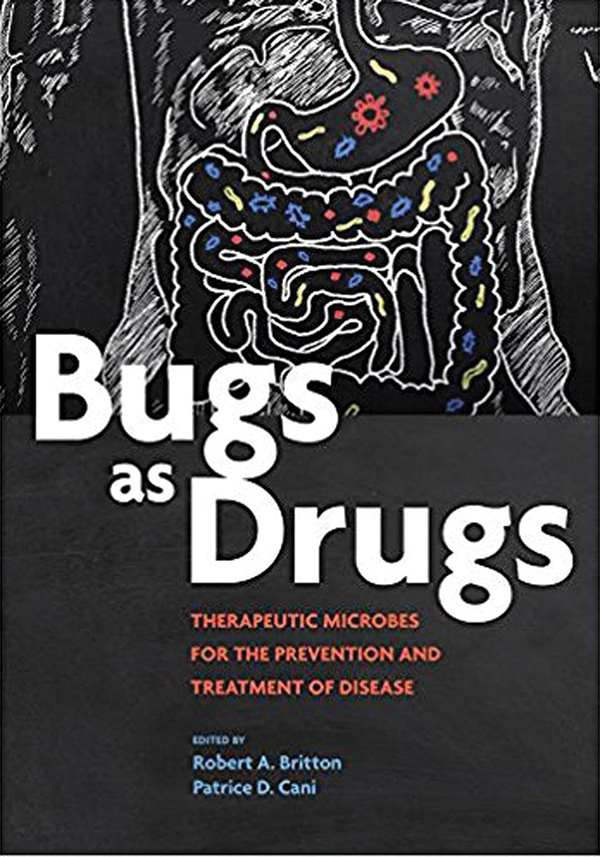
Bugs as Drugs: Therapeutic Microbes for the Prevention and Treatment of Disease

This ≈500-page volume, edited by Robert A. Britton and Patrice D. Cani, comprises 5 main sections of varying scope and length. The introductory section reviews pathways used by commensals, particularly *Lactobacilli* and *Bifidobacteria* spp*., *to benefit the host. The first chapter is particularly impressive, providing a thorough and well-cited overview of the various microbial metabolites and how they influence the host.

The theme of section 2, the longest and most wide-ranging part, is the role of the microbiome in chronic diseases. Given the ambitious scope of this section, the authors selectively covered a range of diseases (from nutritional disorders to colorectal cancer and osteoporosis) for which the host–microbe interactions have been described. Although this section might seem fragmented, it succeeds in providing the reader with a sense of the breadth of diseases in which therapeutic microbes might play a role. When links between microbes or the microbiome and human ailments are tentative or controversial, the authors do a sound job describing the conflicting reports within their respective fields.

Section 3 covers the vital function of the gut microbiome, namely the control of infectious diseases, with a focus on *Clostridioides difficile* and *Enterococcus *spp. infections. In this section, the authors tackle the seminal studies of fecal microbiota transplantation to control *C. difficile* infections and questions that need to be answered to evaluate the long-term consequences of this therapy, extending beyond *C. difficile* control.

Section 4 provides insight on the tools and techniques used to study and manipulate the microbiome, including strategies to genetically modify microbiota to design diagnostics and therapeutic applications, innovative genetic tools to engineer probiotics, and CRISPR (clustered regularly interspaced short palindromic repeats) technologies to modify microbes. Moreover, a chapter in this section is devoted to reviewing US regulatory considerations for developing live biotherapeutics-based drugs; however, regulatory constraints outside the United States are not covered in the book. The final section covers indirect strategies to functionally target the gut microbiome, including the use of bacteriophages as antibacterial agents and prebiotics to modulate the microbiome.

Overall, this book is a commendable and timely volume of well-sourced reviews written by experts in the field well organized into broad research themes. This work will serve as a helpful resource for both scientists and clinicians interested in using microbes for therapeutic applications.

